# The TyG Index, Dietary Vitamin E Intake, and LRP5 rs4988321 in Adults with MASLD: A Case–Control Study

**DOI:** 10.3390/nu18183065

**Published:** 2026-09-19

**Authors:** Kardelen Büşra Ege Gündüz, Ceyda Okudu, Songül Güder, İsa Sevindir

**Affiliations:** 1 Department of Nutrition and Dietetics, Halic University, Istanbul 34060, Türkiye; kardelenegegunduz@halic.edu.tr; 2 Department of Molecular Biology and Genetics, Atlas University, Istanbul 34408, Türkiye; 3 Department of Internal Medicine, İstanbul Training and Research Hospital, Istanbul 34098, Türkiye; songulguder22@gmail.com; 4 Department of Gastroenterology, Medicana Zincirlikuyu Hospital, Istanbul 34394, Türkiye; sevindir@gmail.com

**Keywords:** insulin resistance, triglyceride-glucose index, nutrigenetics, hepatic steatosis, body composition

## Abstract

**Background/Objectives:** Metabolic dysfunction-associated steatotic liver disease (MASLD) is related to insulin resistance, dyslipidemia, and oxidative stress. Vitamin E and low-density lipoprotein receptor-related protein 5 (LRP5) may be involved, but evidence remains limited. This study compared dietary vitamin E intake and *LRP5* rs4988321 genotype distributions between adults with and without MASLD. **Methods:** This single-center case–control study included 59 adults with ultrasonographically confirmed MASLD and 47 controls. Vitamin E intake was assessed using two non-consecutive 24-h dietary recalls and a vitamin E-focused food frequency questionnaire. The rs4988321 variant was genotyped using a TaqMan assay. Group comparisons and multivariable models were used. **Results:** Vitamin E intake did not differ between groups using 24-h recalls (11.81 vs. 9.35 mg/day; *p* = 0.246) or the FFQ (16.47 vs. 17.58 mg/day; *p* = 0.834); only the recall-derived estimate was energy-adjusted and remained unassociated with MASLD. The rs4988321 genotype distributions were similar (*p* = 0.548). After adjustment for age, sex, genotype, and BMI, ALT, LDL cholesterol, and triglyceride levels were nominally higher in participants with MASLD. However, only ALT remained significant after false discovery rate correction (*q* = 0.021). In the BMI-adjusted logistic model, higher TyG index (OR = 2.360; 95% CI: 1.067–5.218; *p* = 0.034) and BMI (OR = 1.304; 95% CI: 1.108–1.535; *p* = 0.001) were associated with greater odds of MASLD. **Conclusions:** Dietary vitamin E intake and the rs4988321 genotype were not associated with MASLD status in this sample. ALT remained higher after multivariable adjustment, whereas the TyG and BMI associations were specific to the BMI-adjusted model.

## 1. Introduction

Metabolic dysfunction-associated steatotic liver disease (MASLD), formerly termed non-alcoholic fatty liver disease, is defined by hepatic steatosis accompanied by at least one cardiometabolic risk factor [1]. Its global burden has increased in parallel with obesity, insulin resistance, and type 2 diabetes, with prevalence estimates rising to approximately 38% in 2016–2019 [2]. MASLD encompasses a spectrum ranging from isolated steatosis to metabolic dysfunction-associated steatohepatitis, progressive fibrosis, cirrhosis, and hepatocellular carcinoma. Insulin resistance, adipose tissue dysfunction, increased hepatic fatty acid delivery, and de novo lipogenesis promote intrahepatic triglyceride accumulation, whereas oxidative stress and lipid peroxidation contribute to hepatocellular injury, inflammation, and fibrogenesis [3].

Vitamin E, particularly α-tocopherol, is a major lipid-soluble, chain-breaking antioxidant that protects polyunsaturated fatty acids in cellular membranes against lipid peroxidation [4]. This antioxidant function provides a biological rationale for its potential role in MASLD, in which increased reactive oxygen species and lipid peroxidation are implicated in disease progression. Vitamin E supplementation has shown potential to improve aminotransferase levels and selected histological features in patients with fatty liver disease [5]. However, existing evidence concerns pharmacological supplementation, and the relationship between habitual dietary vitamin E intake and the clinical and metabolic characteristics of MASLD remains less clearly defined.

Genetic differences in metabolic signaling pathways may further contribute to the heterogeneous phenotype of MASLD. The canonical Wnt/β-catenin pathway, activated through Frizzled receptors and the low-density lipoprotein receptor-related protein 5/6 (LRP5/6) co-receptors, regulates hepatic metabolism, adipose tissue biology, and cellular differentiation. Experimental evidence indicates that Wnt/β-catenin activation can aggravate hepatic steatosis by stimulating fatty acid synthesis through Akt/mTOR signaling and by reducing fatty acid oxidation [6]. Moreover, recent clinical and in vitro findings suggest that LRP5 has metabolic functions beyond bone homeostasis, including the regulation of lower-body fat distribution, adipose progenitor cell fitness, and systemic and adipocyte insulin sensitivity [7].

The LRP5 rs4988321 variant, located in exon 9, is a missense variant that results in a valine-to-methionine substitution at position 667 (p.Val667Met). This variant has been associated with reduced bone mineral density and impaired bone matrix properties, while experimental models have demonstrated alterations in osteoblast differentiation and mineralization [8]. Homozygous carriers of rs4988321, considered a presumed loss-of-function *LRP5* variant, have shown nominally higher insulin resistance indices and triglyceride levels, together with lower leg fat mass, compared with matched controls [7]. Therefore, this study primarily aimed to compare dietary vitamin E intake and LRP5 rs4988321 genotype distribution between adults with and without MASLD. Secondary analyses examined metabolic and anthropometric differences between the groups, while genotype-related biochemical and anthropometric associations were considered exploratory.

## 2. Materials and Methods

### 2.1. Study Design and Participants

This single-center case–control study used cross-sectional clinical, dietary, anthropometric, biochemical, and genetic assessments. Participants were recruited from the Gastroenterology and Internal Medicine clinics of Istanbul Training and Research Hospital between August 2023 and September 2025. The case group comprised adults aged 18–65 years with ultrasonographically confirmed MASLD. The control group comprised adults referred by physicians who were considered not to have hepatic steatosis based on clinical evaluation and review of available biochemical findings and imaging records. However, the control participants did not undergo a standardized study-specific ultrasonographic examination. MASLD was defined as ultrasonographically detected hepatic steatosis together with at least one of the assessed cardiometabolic risk factors: BMI ≥ 25 kg/m^2^ or waist circumference > 94 cm in men and >80 cm in women; fasting plasma glucose ≥ 100 mg/dL, HbA1c ≥ 5.7%, a diagnosis of type 2 diabetes, or antidiabetic treatment; triglycerides ≥ 150 mg/dL or lipid-lowering treatment; or HDL cholesterol ≤ 40 mg/dL in men and ≤50 mg/dL in women or lipid-lowering treatment.

Eligible participants reported alcohol consumption below 30 g/day for men and 20 g/day for women and provided written informed consent. Exclusion criteria were alcohol consumption above these thresholds; alcohol-related fatty liver disease; chronic viral hepatitis; autoimmune hepatitis; hemochromatosis; Wilson’s disease; alpha-1 antitrypsin deficiency; celiac disease; parenteral nutrition; corticosteroid or antiretroviral therapy; previous major abdominal surgery; pregnancy; and lactation.

**Figure 1:**
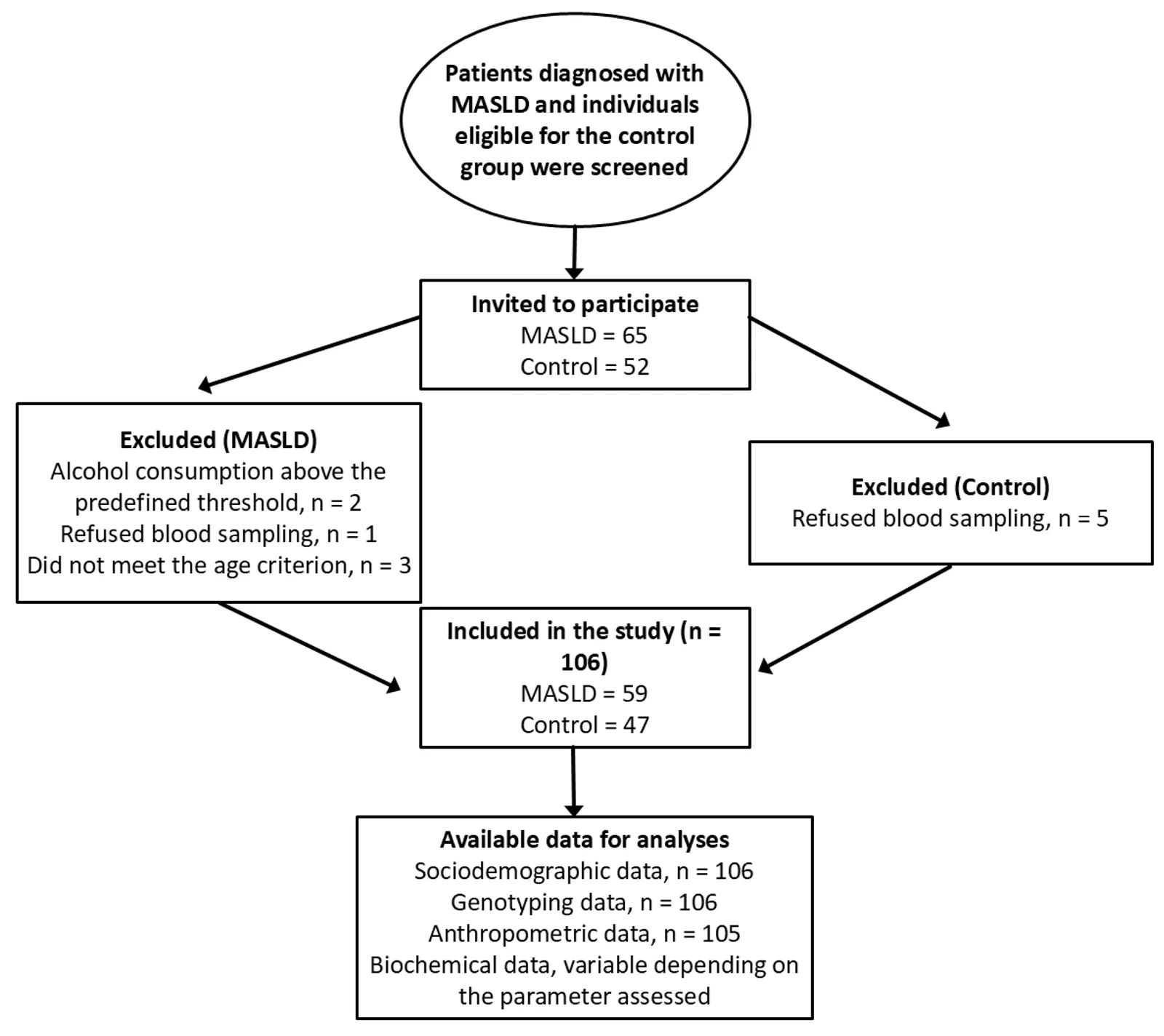
Flow diagram of participant selection and inclusion in the study.

Because comparable prior data were unavailable, an anticipated standardized effect size of 0.60 was used. With α = 0.05 and 80% power, at least 45 participants per group were required. This calculation addressed a general two-group comparison and was not designed to ensure adequate power for either the genetic analyses or the multivariable logistic regression models; therefore, the genetic analyses were considered exploratory. Of 117 individuals invited to participate (65 with MASLD and 52 controls), six potential MASLD participants and five controls were excluded or declined blood sampling. The final sample consisted of 106 participants (59 with MASLD and 47 controls). Participant selection is summarized in Figure 1.

### 2.2. Clinical and Dietary Data Collection

A structured questionnaire was administered by the researchers during a face-to-face interview. It covered sociodemographic characteristics, general health status, smoking and alcohol use, and vitamin or mineral supplement use. Routine biochemical results were extracted from medical records. Variables used in the present analysis included fasting plasma glucose, glycated hemoglobin (HbA1c), high-density lipoprotein cholesterol (HDL), low-density lipoprotein cholesterol (LDL), total cholesterol, triglycerides (TG), aspartate aminotransferase (AST), alanine aminotransferase (ALT), alkaline phosphatase (ALP), and gamma-glutamyl transferase (GGT).

To reduce interviewer-related and measurement variability, questionnaire administration and anthropometric measurements were performed using standardized procedures. Standardized food portion photographs were used to reduce errors in portion-size estimation, and dietary intake was assessed using two non-consecutive 24-h dietary recalls to account for day-to-day variation. The mean intake calculated from the two recalls was used in the analyses. Portion sizes were estimated using the Food and Meal Photograph Catalogue [9]. In addition, a vitamin E-focused food frequency questionnaire (FFQ) comprising commonly consumed vitamin E-rich foods was administered to estimate habitual vitamin E intake. Dietary data from both methods were analyzed with the Computer-Assisted Nutrition Program, Nutrition Information Systems (BEBİS, Mavi Elma Group, Istanbul, Turkiye), and vitamin E intake was expressed as mg/day. Vitamin E adequacy (%) was calculated by dividing daily vitamin E intake by the sex-specific recommendation of the Türkiye Dietary Guidelines 2022 (13 mg/day for men and 11 mg/day for women) and multiplying by 100 [10]. Medication and dietary supplement use were recorded. None of the participants reported using multivitamin or vitamin E-containing supplements; therefore, the calculated vitamin E intake represented dietary sources only.

### 2.3. Anthropometric Measurements and Derived Indices

Anthropometric measurements were performed by the researcher using standardized procedures [11]. Body weight was measured to the nearest 0.1 kg with a digital scale while participants wore light clothing, and standing height was measured without shoes using a stadiometer. Waist circumference was measured midway between the lowest rib and iliac crest, and hip circumference was measured at the widest point of the buttocks. BMI was calculated as weight in kilograms divided by height in metres squared, and the waist-to-hip ratio was calculated by dividing waist circumference by hip circumference.

Weight adjusted waist index (WWI) was calculated as waist circumference (cm) divided by the square root of body weight (kg) [12]. The TyG index was calculated as ln [fasting triglycerides (mg/dL) × fasting glucose (mg/dL)/2] and was used as a surrogate marker of insulin resistance [13].

### 2.4. DNA Extraction and LRP5 rs4988321 Genotyping

Venous blood samples (7–8 mL) were collected into EDTA tubes and sent to Haliç University on the same day at 4 °C for molecular examination. Genomic DNA was extracted from the peripheral blood by salting out method [14].

For LRP5 rs4988321 polymorphism, a probe-based SNP genotyping assay (NucleoGene Probe SNP Assay, ref. NGOT009; NucleoGene Biotechnology, Istanbul, Türkiye) was used with TaqMan^®^ 5′-nuclease chemistry. The assay consisted of primers specific to sequences and two allele-specific MGB probes labeled with the FAM™ and HEX™ fluorophores. PCR reactions were carried out in 20 µL total volume with 5 µL of 4× SNP assay oligonucleotide mix, 10 µL of 2× qPCR Probe Master Mix, 1–20 ng genomic DNA and nuclease-free water. Amplification was performed using an AGS4800 Real-Time PCR System, with an initial denaturation step at 95 °C for 2 min 30 s followed by 40 cycles at 95 °C for 15 s and 60 °C for 60 s.

Genotypes were determined by endpoint allelic discrimination of the fluorescence signals for FAM and HEX. Samples with a predominant signal of one allele-specific probe were classified as homozygous and those with signals from both probes were classified as heterozygous. The genotypes were classified as GG, GA or AA based on allelic discrimination graphs produced by the AGS4800 Software (version 1.1). All samples were genotyped in triplicate, and negative controls were included in each assay. Concordant genotype calls were obtained across replicate measurements.

### 2.5. Statistical Analysis

Statistical analyses were performed using IBM SPSS Statistics version 23.0 and R version 4.6.1. Normality was assessed using the Shapiro–Wilk test. Continuous variables are presented as mean ± standard deviation or median (Q1–Q3), and categorical variables as *n* (%). Groups were compared using the independent-samples *t*-test, Mann–Whitney U test, chi-square test, or Fisher’s exact test, as appropriate. Anthropometric indices were additionally evaluated by sex. Minor allele frequencies and Hardy–Weinberg equilibrium were assessed separately in each group. Because the AA genotype was rare, rs4988321 was analyzed under a dominant model (GA+AA vs. GG). For unadjusted comparisons effect estimates are reported as Hodges–Lehmann median differences with 95% confidence intervals for vitamin E intake and as the unadjusted odds ratio with a 95% Wald confidence interval for the dominant genetic model.

General linear models were used to evaluate biochemical differences according to MASLD status and genotype. For MASLD–control comparisons, adjusted for age, sex, MASLD status, and genotype, as applicable; Model 2 additionally included BMI. The Benjamini–Hochberg procedure was applied separately to MASLD- and genotype-related *p* values across the ten biochemical outcomes within each model, with adjusted values reported as *q* values. Residual normality (Shapiro–Wilk test), homoscedasticity (Breusch–Pagan test), and influential observations (Cook’s distance, using a 4/n screening threshold) were examined for each model, and right-skewed biochemical outcomes (fasting plasma glucose, HbA1c, LDL cholesterol, triglycerides, AST, ALT, ALP, and GGT) were re-analyzed after natural-log transformation as sensitivity analyses. Residual normality and homoscedasticity were re-examined after transformation, and heteroscedasticity-consistent (HC3) standard errors were computed for all log-scale models as an additional sensitivity analysis. The same ten-outcome family was used for the log-scale sensitivity analyses, with the untransformed outcomes (HDL cholesterol and total cholesterol) retained in the family. Multivariable logistic regression using the Enter method was performed to identify factors associated with MASLD. Model 1 included age, sex, FFQ-derived vitamin E adequacy, TyG index, genotype, and WWI; Model 2 additionally included BMI. Logistic model fit was evaluated using the omnibus likelihood-ratio test, the Hosmer–Lemeshow test, Nagelkerke R^2^, and classification indices. Linearity of the logit and multicollinearity were assessed using the Box-Tidwell procedure and variance inflation factors, respectively. Robustness was examined using Firth regression and 1000-replicate bootstrap confidence intervals. Vitamin E intake derived from the two 24-h dietary records was energy-adjusted using the residual method and expressed per 1000 kcal using the nutrient-density method; FFQ-derived vitamin E intake was not energy-adjusted because the FFQ did not provide an estimate of total energy intake. The analyses were repeated among participants not reporting vitamin–mineral supplement use. Exploratory sex × MASLD interactions were examined for anthropometric outcomes. Medication-related sensitivity analyses were performed by adding statin and antidiabetic medication use to the logistic model and by repeating the biochemical comparisons after excluding statin users. The minimum detectable odds ratio for the genetic association was estimated by simulation at 80% power and a two-sided α of 0.05. General linear models were based on available data, whereas logistic regression analyses used complete cases. Missing values were not imputed. Analyses in R version 4.6.1 used the packages car 3.1–5 (variance inflation factors), ResourceSelection 0.3–6 (Hosmer–Lemeshow test), logistf 1.26.1 (Firth regression), boot 1.3–32 (bootstrap), sandwich 3.1–2 and lmtest 0.9–40 (HC3 standard errors), and e1071 1.7–17 (skewness) [15,16,17,18,19,20,21]. Two-sided *p* < 0.05 and, where applicable, q < 0.05 were considered statistically significant.

## 3. Results

### 3.1. Characteristics of Participants

The MASLD group comprised 39 women (66.1%) and 20 men (33.9%), and the control group comprised 29 women (61.7%) and 18 men (38.3%), with no significant difference in sex distribution between the groups (*p* = 0.639). Participants with MASLD were older than controls (46.34 ± 8.91 vs. 36.89 ± 13.19 years, *p* < 0.001). Glucose (*p* < 0.001), HbA1c (*p* = 0.028), LDL cholesterol (*p* = 0.004), total cholesterol (*p* = 0.002), triglycerides (*p* = 0.002), AST (*p* = 0.018), ALT (*p* < 0.001), and GGT (*p* = 0.010) were significantly higher in the MASLD group, whereas HDL cholesterol (*p* = 0.917) and ALP (*p* = 0.155) did not differ significantly between the groups. Among women, BMI, WWI, the waist-to-hip ratio, and the TyG index were significantly higher in the MASLD group (*p* < 0.001, *p* < 0.001, *p* < 0.001, and *p* = 0.009, respectively). Among men, BMI (*p* = 0.001) and the TyG index (*p* = 0.021) were significantly higher in the MASLD group, whereas WWI (*p* = 0.491) and the waist-to-hip ratio (*p* = 0.173) did not differ significantly (Table 1). Elevated BMI or waist circumference was present in 55 participants with MASLD (93.2%) and 21 controls (44.7%); impaired glucose metabolism was present in 36 (61.0%) and 16 (34.0%); elevated triglycerides were present in 26 (44.1%) and 10 (21.3%); and low HDL cholesterol was present in 21 (35.6%) and 16 (34.0%), respectively.

### 3.2. Dietary Vitamin E Intake and rs4988321 Genotype Distribution

Median vitamin E intake did not differ significantly between the MASLD and control groups, either from the 24-h dietary recalls (11.81 vs. 9.35 mg/day, *p* = 0.246) or from the FFQ (16.47 vs. 17.58 mg/day, *p* = 0.834). The GG genotype was observed in 48 participants (81.4%) in the MASLD group and 36 (76.6%) in the control group, whereas the GA+AA genotypes were observed in 11 participants in each group (18.6% and 23.4%, respectively), with no significant difference under the dominant genetic model (*p* = 0.548). The minor allele frequency was 0.093 in the MASLD group and 0.128 in the control group, and genotype distributions were consistent with the Hardy–Weinberg equilibrium in both groups (*p* = 0.429 and *p* = 0.759, respectively). The Hodges–Lehmann median difference (MASLD minus control) was 1.35 mg/day (95% CI: −1.07 to 3.46) for 24-h recall intake and −0.23 mg/day (95% CI: −4.21 to 4.27) for FFQ-derived intake, and the unadjusted odds ratio for MASLD in GA+AA versus GG carriers was 0.75 (95% CI: 0.29–1.92) (Table 2).

### 3.3. Biochemical Comparisons According to MASLD Status

In the adjusted analyses, ALT, triglyceride, and LDL cholesterol levels were higher in participants with MASLD in Model 1. After false discovery rate correction, all three differences remained significant (q = 0.006, q = 0.036, and q = 0.036, respectively). In Model 2, which additionally included BMI, ALT, triglyceride, and LDL cholesterol remained nominally higher in participants with MASLD; however, only the ALT difference remained significant after false discovery rate correction (q = 0.021) (Table 3).

In the log-transformed sensitivity analyses (Supplementary Table S1), the MASLD-associated differences in ALT (geometric mean ratio [GMR]: 1.753, 95% CI: 1.375–2.234, *p* < 0.001), LDL cholesterol (GMR: 1.254, 95% CI: 1.056–1.489, *p* = 0.010), and triglycerides (GMR: 1.437, 95% CI: 1.099–1.877, *p* = 0.008) remained significant in Model 1 after false discovery rate correction (q < 0.001, q = 0.035, and q = 0.035, respectively), whereas in Model 2 only the ALT difference did (q < 0.001; LDL cholesterol, *p* = 0.015, q = 0.074; triglycerides, *p* = 0.053, q = 0.134). The AST difference was nominally significant on the log scale in Model 1 (GMR: 1.259, 95% CI: 1.037–1.527, *p* = 0.020) and Model 2 (GMR: 1.251, 95% CI: 1.009–1.550, *p* = 0.041) but did not remain significant after correction (q = 0.050 and q = 0.134, respectively). After log transformation, residual normality was acceptable for ALT, LDL cholesterol, and triglycerides (Shapiro–Wilk *p* ≥ 0.05), and homoscedasticity was acceptable for all transformed outcomes except AST in Model 2 (Breusch–Pagan *p* = 0.044), whereas residual non-normality persisted for glucose, HbA1c, AST, ALP, and GGT despite markedly reduced skewness. With heteroscedasticity-consistent (HC3) standard errors, the conclusions for ALT, LDL cholesterol, and triglycerides were unchanged; the AST difference remained significant in Model 1 (*p* = 0.044) but not in Model 2 (*p* = 0.086), and the glucose difference in Model 1 reached nominal significance (*p* = 0.048) without doing so in any other specification (Supplementary Table S1). After excluding statin users, the LDL cholesterol difference remained significant (β = 21.01; *p* = 0.039), whereas the triglyceride difference was no longer significant (β = 5.06; *p* = 0.744).

### 3.4. Biochemical Comparisons According to the LRP5 rs4988321 Genotype

In the analyses based on the dominant genetic model, GA+AA carriers had nominally higher ALT levels than GG homozygotes in Model 1 (β = 12.79; 95% CI: 1.35–24.23; *p* = 0.029) and Model 2 (β = 12.81; 95% CI: 1.24–24.39; *p* = 0.030). However, these differences did not remain significant after false discovery rate correction (q = 0.288 and q = 0.304, respectively) or in the log-transformed sensitivity analysis. (GMR: 1.250, 95% CI: 0.947–1.650, q = 0.566 in Model 1; GMR: 1.243, 95% CI: 0.939–1.645, q = 0.635 in Model 2; Supplementary Table S1). No statistically significant genotype-related differences were observed for the other biochemical parameters (Table 4).

### 3.5. Factors Associated with MASLD

In the multivariable logistic regression analysis, the TyG index was not significantly associated with MASLD in Model 1 (OR = 1.908; 95% CI: 0.946–3.849; *p* = 0.071). After BMI was added, higher TyG index (OR = 2.360; 95% CI: 1.067–5.218; *p* = 0.034) and BMI (OR = 1.304; 95% CI: 1.108–1.535; *p* = 0.001) were associated with greater odds of MASLD. Age, sex, FFQ-derived vitamin E adequacy, genotype, and WWI were not significant in either model (Table 5).

Both logistic models were based on 90 complete cases and were statistically significant overall (both *p* < 0.001). The Hosmer–Lemeshow test indicated adequate calibration for Models 1 and 2 (*p* = 0.173 and *p* = 0.140), with Nagelkerke R^2^ values of 0.328 and 0.467 and classification accuracies of 73.3% and 77.8%, respectively. No problematic multicollinearity was detected (all VIF ≤ 1.95). Models including a quadratic age term, Firth regression, and bootstrap analyses did not materially change the findings. Energy-adjusted vitamin E intake remained unassociated with MASLD using the residual method (*p* = 0.750 and *p* = 0.993) and the nutrient-density method (*p* = 0.702 and *p* = 0.500) in Models 1 and 2, respectively. Available and missing observations are presented in Supplementary Table S2. No age-adjusted sex × MASLD interaction was statistically significant; full estimates are presented in Supplementary Table S3. Further adjustment for statin and antidiabetic medication use did not materially alter the Model 2 findings for the TyG index (OR = 2.476; 95% CI: 1.005–6.101; *p* = 0.049) or BMI (OR = 1.300; 95% CI: 1.103–1.533; *p* = 0.002). The minimum detectable odds ratio for the genetic association was 5.45.

## 4. Discussion

The main findings of this study were that dietary vitamin E intake and LRP5 rs4988321 genotype distributions did not differ between participants with and without MASLD. Among the biochemical variables, ALT showed the most consistent difference between the groups. The TyG index and BMI were associated with MASLD in the BMI-adjusted logistic regression model. In contrast, no clear association was found between WWI or rs4988321 and MASLD.

Insulin resistance promotes the release of free fatty acids from adipose tissue, increases hepatic de novo lipogenesis, and enhances the production of triglyceride-rich lipoproteins. These processes contribute to both intrahepatic triglyceride accumulation and the dyslipidemic profile associated with MASLD [3,22]. Current EASL-EASD-EASO guidance similarly emphasizes the close relationship of MASLD with insulin resistance, dyslipidemia, obesity, and cardiovascular risk [22]. In the present study, ALT, triglyceride, and LDL cholesterol differences remained significant after false discovery rate correction in Model 1. However, after additional adjustment for BMI, only the ALT difference remained significant. LDL cholesterol remained significant in the analysis excluding statin users, whereas the triglyceride difference was attenuated. Therefore, the triglyceride finding should be interpreted cautiously.

ALT remained higher in the MASLD group after adjustment for BMI and false discovery rate correction. The difference also remained evident in the log-transformed analysis. Although ALT may reflect hepatocellular injury, it is neither a direct measure of hepatic fat content nor a sufficiently sensitive marker of steatohepatitis or fibrosis. Normal aminotransferase concentrations do not exclude MASLD or advanced liver disease, while elevated concentrations cannot establish histological severity [22]. Accordingly, the present ALT findings should not be interpreted as evidence of MASH or advanced fibrosis. The AST difference was nominally significant only in the log-transformed sensitivity analysis but did not remain significant after false discovery rate correction; it should therefore be interpreted cautiously.

Triglycerides and aminotransferases may be responsive to dietary modification. A recent systematic review and meta-analysis of three randomized trials involving 220 adults with NAFLD reported that canola oil consumption was associated with modest reductions in triglycerides, ALT, and AST, whereas no significant effects were observed for HDL cholesterol or GGT [23]. Although these intervention findings are not directly comparable with the present observational results, they highlight the potential relevance of dietary fat quality to selected hepatic and cardiometabolic markers.

The TyG index was not significantly associated with MASLD in Model 1. After BMI was added to the model, higher TyG index and BMI were both associated with greater odds of MASLD. The TyG index combines fasting triglyceride and glucose concentrations and was originally proposed as a practical surrogate marker of insulin resistance [24]. Previous meta-analytic evidence has demonstrated a positive association between the TyG index and fatty liver disease, although heterogeneity in disease definitions and TyG thresholds limits the establishment of a universal clinical cut-off [25]. In the present study, the TyG association was observed only after BMI was included in the model. This model-dependent finding may reflect the interrelationships among adiposity, glucose metabolism, and triglyceride concentrations and does not, by itself, demonstrate that the TyG index provides predictive information beyond BMI. Therefore, this association should be considered exploratory.

WWI was originally developed to standardize waist circumference relative to body weight and to reflect abdominal adiposity while reducing the strong dependence of waist circumference on overall body size [12]. Population-based studies have subsequently reported positive associations between WWI and NAFLD [26]. WWI was not statistically associated with MASLD after multivariable adjustment in the present study. The difference observed among women was supported by an unadjusted sex-by-MASLD interaction for WWI, but this interaction was attenuated after adjustment for age. This finding should therefore be considered exploratory.

Neither 24-h recall-derived nor FFQ-derived vitamin E intake differed significantly between the groups. Vitamin E also remained unassociated with MASLD after adjustment for total energy intake using both the residual and nutrient-density methods. Similar results were obtained when the analyses were restricted to participants not reporting vitamin–mineral supplement use. These findings indicate that the absence of an association was consistent across different analytical approaches. Previous research in pediatric NAFLD associated lower vitamin E intake with more severe hepatic steatosis, although differences in population characteristics and disease assessment limit direct comparison with the present adult sample [27]. Furthermore, dietary vitamin E intake should not be considered equivalent to circulating or tissue vitamin E status. Previous studies have reported altered plasma vitamin E concentrations in NASH, demonstrating that intake data and biochemical vitamin E status may provide different information [28]. The lack of serum or lipid-adjusted α-tocopherol measurements in the present study therefore limits conclusions regarding biological vitamin E status.

Recent population-based evidence has associated higher dietary vitamin E intake among individuals with MASLD with lower subsequent all-cause and cardiovascular mortality [29]. This does not necessarily conflict with the absence of an association with MASLD status in the present study because disease prevalence and long-term prognosis represent different outcomes. Likewise, the biochemical and histological benefits reported for pharmacological vitamin E supplementation [5] cannot be extrapolated directly to habitual dietary vitamin E intake.

A previous study examining another LRP5 variant, rs556442, reported an association with NAFLD among individuals with coronary heart disease; however, no significant difference was observed between the overall NAFLD and control groups [30]. These findings cannot be directly extrapolated to rs4988321 because the two variants may have different functional effects and allele distributions. LRP5 is a co-receptor in the canonical Wnt/β-catenin pathway and contributes to cholesterol metabolism and glucose-stimulated insulin secretion [31]. Experimental evidence also indicates that activation of hepatic Wnt/β-catenin signalling may promote lipogenesis through interactions with the Akt/mTOR pathway [6]. The rs4988321 variant causes a p.Val667Met substitution, and functional evidence derived mainly from bone-related models suggests that this variant may compromise canonical Wnt signalling [8]. These findings provide biological plausibility for metabolic effects but do not establish a liver-specific mechanism.

GA+AA carriers initially appeared to have higher ALT levels than GG homozygotes. However, this difference did not remain significant after false discovery rate correction or log transformation. It should therefore be regarded as a nominal and exploratory finding rather than evidence of a genotype-related effect on liver injury. Moreover, the minimum detectable odds ratio was 5.45, showing that the study had sufficient power to detect only large genetic effects. Thus, the findings do not exclude smaller associations between rs4988321 and MASLD.

This study has several strengths. It provides an integrated evaluation of dietary, genetic, anthropometric, and biochemical factors in adults with and without MASLD. Vitamin E intake was assessed using two complementary dietary assessment methods, allowing both recent and habitual intake to be considered. Genotyping was performed using a standardized TaqMan assay. The additional energy-adjusted, sensitivity, and false discovery rate analyses also allowed the consistency of the findings to be evaluated.

This study should be interpreted in light of several limitations. First, its cross-sectional case–control design does not allow temporal or causal relationships to be established. Second, the single-center setting and modest sample size may limit the generalizability of the findings. The low frequency of the rs4988321 minor allele, together with the absence of AA carriers in the MASLD group, reduced the precision of the genetic estimates and prevented the evaluation of alternative genetic models. The power analysis also indicated that smaller genetic effects could not be reliably detected. Third, hepatic steatosis was assessed by ultrasonography, which may be less sensitive for mild steatosis and cannot characterize steatohepatitis or fibrosis. Although control participants were selected by physicians following evaluation of available clinical, biochemical, and imaging records, they did not undergo a uniform study-specific ultrasonographic examination. Because controls did not undergo uniform study-specific ultrasonography, some participants with unrecognized mild steatosis may have been misclassified as controls. This potential misclassification could have attenuated between-group differences and biased the observed associations toward the null, particularly for the primary vitamin E and rs4988321 comparisons. Because cardiometabolic risk factors form part of the diagnostic definition of MASLD, the observed associations of BMI and the TyG index with MASLD status may partly reflect overlap with the metabolic features used to define the disease. Therefore, these associations should be interpreted as descriptive and should not be considered evidence of independent etiological or predictive effects. Direct blood pressure measurements and detailed antihypertensive treatment data were not available, so this cardiometabolic criterion could not be fully evaluated. HbA1c was unavailable for 39 participants, and the reason for these missing measurements was not recorded. Although medication-related sensitivity analyses were performed, residual confounding related to medication use cannot be excluded. Physical activity and socioeconomic status were not included in the multivariable models, and residual confounding related to these factors cannot be excluded. In addition, despite the application of false discovery rate correction to biochemical models, the large number of exploratory comparisons may have increased the possibility of chance findings.

Finally, dietary vitamin E intake was based on self-reported assessments and may have been affected by recall and measurement errors. The vitamin E-focused FFQ was not independently validated and did not provide total energy intake; therefore, energy adjustment could only be applied to the 24-h dietary recalls. Circulating α-tocopherol concentrations were also not measured. Larger, multicenter prospective studies incorporating quantitative liver assessments, vitamin E biomarkers, and larger numbers of minor-allele carriers are needed to confirm these findings.

## 5. Conclusions

Dietary vitamin E intake was not associated with MASLD, including after adjustment for total energy intake. No association was detected between LRP5 rs4988321 genotype and MASLD status in this sample; however, the limited number of minor-allele carriers and low statistical power prevented smaller genetic effects from being excluded. ALT showed the most consistent adjusted difference between participants with MASLD and controls, although this finding should not be interpreted as evidence of MASH or fibrosis. Higher TyG index and BMI were associated with greater odds of MASLD only in the BMI-adjusted model; therefore, these model-dependent findings should be interpreted cautiously. Larger prospective studies using quantitative liver assessments, biochemical markers of vitamin E status, and larger numbers of minor-allele carriers are needed to confirm these findings.

## Figures and Tables

**Table 1:** Clinical, biochemical, and anthropometric characteristics of the MASLD and control groups.

Variable	MASLD *n* (%)	Control *n* (%)	Total *n* (%)	*p* Value
**Sex**				
Female	39 (66.1)	29 (61.7)	68 (64.2)	0.639
Male	20 (33.9)	18 (38.3)	38 (35.8)	
**Variable**	**MASLD**	**Control**	***p* value**	
Age (years), mean ± SD (min-max)	46.34 ± 8.91 (25–65)	36.89 ± 13.19 (21–64)	<0.001 *	
Glucose (mg/dL), median (Q1–Q3)	99 (93–114)	89 (83–100)	<0.001 *	
HbA1c (%), median (Q1–Q3)	5.8 (5.4–6.4)	5.4 (5.1–5.9)	0.028 *	
HDL (mg/dL), mean ± SD (min-max)	51.27 ± 12.54 (26–82)	51.58 ± 16.69 (19–96)	0.917	
LDL (mg/dL), median (Q1–Q3)	132 (99–155)	104 (85–128)	0.004 *	
Total cholesterol (mg/dL), median (Q1–Q3)	215 (178.5–234)	174 (148–206)	0.002 *	
Triglycerides (mg/dL), median (Q1–Q3)	136 (85.5–205.5)	105.5 (54–123)	0.002 *	
AST (U/L), median (Q1–Q3)	21 (18–30)	17.5 (14–28)	0.018 *	
ALT (U/L), median (Q1–Q3)	23 (16–35)	14 (11–19.3)	<0.001 *	
ALP (U/L), median (Q1–Q3)	69.2 (58.7–101)	66.5 (50–84.1)	0.155	
GGT (U/L), median (Q1–Q3)	24 (17–35.85)	15.15 (10–27.1)	0.010 *	
**BMI (kg/m^2^)**				
Women	31.33 (28.80–34.51)	23.90 (21.64–30.12)	<0.001 *	
Men	29.88 ± 3.98 (23.5–38.1)	25.04 ± 4.00 (16.82–32.19)	0.001 *	
**WWI**				
Women	11.33 (10.68–11.72)	9.88 (9.71–10.78)	<0.001 *	
Men	11.04 ± 0.81 (9.64–12.39)	10.85 ± 0.83 (9.52–12.78)	0.491	
**Waist-to-hip ratio**				
Women	0.89 ± 0.06 (0.74–1.03)	0.80 ± 0.06 (0.72–0.95)	<0.001 *	
Men	0.96 ± 0.08 (0.77–1.07)	0.92 ± 0.08 (0.81–1.15)	0.173	
**TyG index**				
Women	8.84 ± 0.72 (7.58–10.11)	8.31 ± 0.77 (7.13–10.02)	0.009 *	
Men	9.08 ± 0.78 (7.88–10.78)	8.45 ± 0.64 (7.62–9.42)	0.021 *	

Data are presented as mean ± SD (min-max), median (Q1–Q3), or n (%), as appropriate. Continuous variables were compared using the independent-samples *t*-test or Mann–Whitney U test, and categorical variables using the chi-square test or Fisher’s exact test, as appropriate. *p* < 0.05 was considered statistically significant. * Statistically significant.

**Table 2:** Dietary vitamin E intake and LRP5 rs4988321 genotype distribution in the MASLD and control groups.

Variable	MASLD	Control	Effect Estimate (95% CI)	*p* Value
Dietary vitamin E intake				
24-h recall vitamin E intake (mg/day), median (Q1–Q3)	11.81 (8.49–15.70)	9.35 (6.66–14.73)	1.35 (−1.07 to 3.46)	0.246
FFQ-derived vitamin E intake (mg/day), median (Q1–Q3)	16.47 (12.01–24.36)	17.58 (9.78–23.44)	−0.23 (−4.21 to 4.27)	0.834
LRP5 rs4988321 genotype distribution, *n* (%)				
GG	48 (81.4)	36 (76.6)		
GA	11 (18.6)	10 (21.3)		
AA	0(0.0)	1 (2.1)		
GG vs. GA+AA	48 (81.4) vs. 11 (18.6)	36 (76.6) vs. 11 (23.4)	0.75 (0.29–1.92)	0.548
MAF	0.093	0.128		
HWE *p* value	0.429	0.759		

FFQ, food frequency questionnaire, MAF, minor allele frequency; HWE, Hardy–Weinberg equilibrium. Continuous variables are presented as median (Q1–Q3), and categorical variables are presented as n (%). Between-group comparisons for dietary vitamin E intake were performed using the Mann–Whitney U test. Genotype distributions were compared using the chi-square test or Fisher’s exact test, as appropriate. The GG vs. GA+AA comparison represents the dominant genetic model. Effect estimates are Hodges–Lehmann median differences (MASLD minus control) with 95% confidence intervals for vitamin E intake and the unadjusted odds ratio (GA+AA vs. GG) with 95% confidence interval for the dominant genetic model.

**Table 3:** Biochemical parameters according to MASLD status and adjusted between-group comparisons.

Parameter	MASLD, Unadjusted Value	Control, Unadjusted Value	Unadjusted *p*	Model 1: MASLD−Control Difference (95% CI) †	*p*	*q*	Model 2: MASLD−Control Difference (95% CI) ‡	*p*	*q*
**Fasting plasma glucose (mg/dL), median (Q1–Q3)**	99 (93–114), n = 59	89 (83–100), n = 44	<0.001 *	12.92 (−8.17–34.00)	0.227	0.378	12.20 (−11.34–35.73)	0.306	0.510
**HbA1c (%), median (Q1–Q3)**	5.8 (5.4–6.4), n = 37	5.4 (5.1–5.9), n = 30	0.028 *	0.31 (−0.72–1.33)	0.554	0.791	0.01 (−1.14–1.17)	0.981	0.981
**HDL cholesterol (mg/dL), mean ± SD (min-max)**	51.27 ± 12.54 (26–82), n = 53	51.58 ± 16.69 (19–96), n = 42	0.917	−0.74 (−7.14–5.67)	0.819	0.964	−0.49 (−7.51–6.53)	0.890	0.981
**LDL cholesterol (mg/dL), median (Q1–Q3)**	132 (99–155), n = 51	104 (85–128), n = 42	0.004 *	28.64 (6.79–50.49)	0.011	0.036 *	29.28 (5.48–53.08)	0.017	0.083
**Total cholesterol (mg/dL), median (Q1–Q3)**	215 (178.5–234), n = 53	174 (148–206), n = 41	0.002 *	18.61 (−3.32–40.53)	0.095	0.190	16.68 (−7.27–40.63)	0.170	0.425
**Triglycerides (mg/dL), median (Q1–Q3)**	136 (85.5–205.5), n = 52	105.5 (54–123), n = 42	0.002 *	66.82 (17.66–115.98)	0.008	0.036 *	56.67 (2.97–110.37)	0.039	0.129
**AST (U/L), median (Q1–Q3)**	21 (18–30), n = 59	17.5 (14–28), n = 46	0.018 *	7.07 (−0.04–14.17)	0.051	0.128	4.44 (−3.35–12.24)	0.261	0.510
**ALT (U/L), median (Q1–Q3)**	23 (16–35), n = 59	14 (11–19.3), n = 46	<0.001 *	17.77 (7.76–27.77)	0.001	0.006 *	17.69 (6.58–28.80)	0.002	0.021 *
**ALP (U/L), median (Q1–Q3)**	69.2 (58.7–101), n = 43	66.5 (50–84.1), n = 36	0.155	−1.50 (−20.43–17.44)	0.875	0.964	0.31 (−20.52–21.14)	0.977	0.981
**GGT (U/L), median (Q1–Q3)**	24 (17–35.85), n = 48	15.15 (10–27.1), n = 38	0.010 *	0.42 (−18.10–18.93)	0.964	0.964	6.92 (−13.52–27.37)	0.502	0.718

Unadjusted values are presented as mean ± standard deviation (minimum-maximum) or median (Q1–Q3), as appropriate. Positive adjusted differences indicate higher values in the MASLD group. † MASLD/control comparison adjusted for age, sex, and rs4988321 dominant genotype. ‡ MASLD/control comparison adjusted for Model 1 and BMI. *q*, Benjamini–Hochberg false discovery rate-adjusted *p* value, calculated across the 10 biochemical outcomes separately within each model. * *p* < 0.05. CI, confidence interval; ALT, alanine aminotransferase; AST, aspartate aminotransferase; ALP, alkaline phosphatase; GGT, gamma-glutamyl transferase.

**Table 4:** Adjusted biochemical comparisons according to the LRP5 rs4988321 dominant genetic model.

Parameter	Model 1: GA+AA−GG Difference (95% CI) †	*p*	*q*	Model 2: GA+AA−GG Difference (95% CI) ‡	*p*	*q*
**Fasting plasma glucose (mg/dL)**	−8.06 (−31.78–15.66)	0.502	0.839	−7.88 (−31.86–16.11)	0.516	0.860
**HbA1c (%)**	−0.25 (−1.42–0.92)	0.671	0.839	−0.18 (−1.35–1.00)	0.763	0.953
**HDL cholesterol (mg/dL)**	0.10 (−7.15–7.34)	0.979	0.991	−0.04 (−7.48–7.41)	0.992	0.992
**LDL cholesterol (mg/dL)**	5.35 (−19.48–30.18)	0.669	0.839	5.00 (−20.50–30.49)	0.698	0.953
**Total cholesterol (mg/dL)**	0.14 (−24.93–25.22)	0.991	0.991	1.18 (−24.52–26.88)	0.927	0.992
**Triglycerides (mg/dL)**	17.24 (−38.27–72.76)	0.539	0.839	22.77 (−34.01–79.55)	0.428	0.855
**AST (U/L)**	4.63 (−3.49–12.76)	0.261	0.652	5.35 (−2.77–13.47)	0.194	0.592
**ALT (U/L)**	12.79 (1.35–24.23)	0.029 *	0.288	12.81 (1.24–24.39)	0.030 *	0.304
**ALP (U/L)**	18.03 (−3.15–39.21)	0.094	0.470	17.12 (−4.61–38.85)	0.121	0.592
**GGT (U/L)**	15.55 (−5.66–36.75)	0.148	0.495	12.81 (−8.58–34.21)	0.237	0.592

Positive adjusted differences indicate higher values in the GA+AA group. † Adjusted for age, sex, and MASLD status. ‡ Adjusted for age, sex, MASLD status, and BMI. * *p* < 0.05. *q*, Benjamini–Hochberg false discovery rate-adjusted *p* value calculated across the 10 biochemical outcomes separately within each model. CI, confidence interval; ALT, alanine aminotransferase; AST, aspartate aminotransferase; ALP, alkaline phosphatase; GGT, gamma-glutamyl transferase.

**Table 5:** Factors associated with MASLD in the adjusted models.

Variable	Model 1 B	OR (95% CI)	*p*	Model 2 B	OR (95% CI)	*p*
**Age (years)**	0.053	1.054 (0.998–1.113)	0.058	0.053	1.055 (0.995–1.118)	0.075
**Sex (women vs. men)**	0.638	1.892 (0.668–5.358)	0.230	0.130	1.139 (0.369–3.515)	0.821
**BMI (kg/m^2^)**	—	—	—	0.266	1.304 (1.108–1.535)	0.001 *
**Vitamin E adequacy from the FFQ (%)**	−0.005	0.995 (0.963–1.028)	0.752	−0.001	0.999 (0.965–1.034)	0.946
**TyG index**	0.646	1.908 (0.946–3.849)	0.071	0.858	2.360 (1.067–5.218)	0.034 *
**rs4988321 (GA+AA vs. GG)**	−0.147	0.864 (0.245–3.040)	0.819	0.407	1.502 (0.380–5.940)	0.562
**WWI**	0.476	1.609 (0.741–3.492)	0.229	−0.331	0.718 (0.284–1.818)	0.485

Model 1 included age, sex, vitamin E adequacy estimated from the FFQ, TyG index, the rs4988321 dominant genetic model, and WWI. Model 2 additionally included BMI. Both models were fitted using the Enter method. * *p* < 0.05. BMI, body mass index; CI, confidence interval; FFQ, food frequency questionnaire; OR, odds ratio; TyG, triglyceride-glucose; WWI, weight-adjusted waist index.

## Data Availability

The data presented in this study are available from the corresponding author upon reasonable request. The data are not publicly available due to participant privacy and ethical considerations.

## Supplementary Materials

The following supporting information can be downloaded at https://www.mdpi.com/article/10.3390/nu18183065/s1. Table S1: Log-transformed sensitivity analyses of the biochemical outcomes: geometric mean ratios for MASLD versus control and for GA+AA versus GG carriage, with residual diagnostics after transformation. (A) Model 1; (B) Model 2; Table S2: Available and missing observations for the study variables, by group; Table S3: Sex × MASLD interaction estimates for the anthropometric outcomes (exploratory analyses).

## Abbreviations

The following abbreviations are used in this manuscript:

**Abbreviation**	**Definition**
Akt	Protein kinase B
ALP	Alkaline phosphatase
ALT	Alanine aminotransferase
AST	Aspartate aminotransferase
BEBİS	Nutrition Information System software
BMI	Body mass index
CI	Confidence interval
DNA	Deoxyribonucleic acid
EASD	European Association for the Study of Diabetes
EASL	European Association for the Study of the Liver
EASO	European Association for the Study of Obesity
EDTA	Ethylenediaminetetraacetic acid
FFQ	Food frequency questionnaire
GGT	Gamma-glutamyl transferase
HbA1c	Glycated hemoglobin
HDL	High-density lipoprotein cholesterol
HWE	Hardy–Weinberg equilibrium
LDL	Low-density lipoprotein cholesterol
LRP5	Low-density lipoprotein receptor-related protein 5
LRP6	Low-density lipoprotein receptor-related protein 6
MAF	Minor allele frequency
MASH	Metabolic dysfunction-associated steatohepatitis
MASLD	Metabolic dysfunction-associated steatotic liver disease
mTOR	Mechanistic target of rapamycin
NAFLD	Non-alcoholic fatty liver disease
NASH	Non-alcoholic steatohepatitis
OR	Odds ratio
PCR	Polymerase chain reaction
SD	Standard deviation
SNP	Single-nucleotide polymorphism
TyG	Triglyceride-glucose index
WWI	Weight-adjusted waist index
