# Supplementary material for: The TyG Index, Dietary Vitamin E Intake, and LRP5 rs4988321 in Adults with MASLD: A Case–Control Study
Source: Nutrients. 2026 Sep 19;18(18):3065. doi: 10.3390/nu18183065 (PMC13610090; doi:10.3390/nu18183065)
Supplement: Supplementary file 1 [file nutrients-18-03065-s001.zip › nutrients-4523991-supplementary.pdf]

Supplementary Material for TyG Index, Dietary Vitamin E Intake, and LRP5 rs4988321 in Adults with MASLD: A Case-Control Study

Kardelen Büşra Ege Gündüz[1], Ceyda Okudu[2*], Songül Güder[3] and İsa Sevindir[4]

*Correspondence: ceyda.okudu@atlas.edu.tr

Supplementary Table S1. Log-transformed sensitivity analyses of the biochemical outcomes: geometric mean ratios for MASLD versus control and for GA+AA versus GG carriage, with residual diagnostics after transformation. (A) Model 1; (B) Model 2.

Outcome	MASLD vs. control				GA+AA vs. GG				SW p	BP p
	GMR (95% CI)	p	q	p (HC3)	GMR (95% CI)	p	q	p (HC3)		
**A. Model 1**										
Fasting plasma glucose	1.119 (0.983–1.275)	0.089	0.159	0.048	0.964 (0.833–1.116)	0.625	0.991	0.474	<0.001	0.174
HbA1c	1.047 (0.921–1.190)	0.479	0.684	0.417	0.987 (0.852–1.143)	0.857	0.991	0.761	<0.001	0.230
LDL cholesterol	1.254 (1.056–1.489)	0.010	0.035	0.021	1.076 (0.885–1.307)	0.459	0.919	0.363	0.053	0.521
Triglycerides	1.437 (1.099–1.877)	0.008	0.035	0.032	0.962 (0.711–1.301)	0.799	0.991	0.819	0.108	0.380
AST	1.259 (1.037–1.527)	0.020	0.050	0.044	1.109 (0.889–1.384)	0.353	0.919	0.345	0.002	0.925
ALT	1.753 (1.375–2.234)	<0.001	<0.001	<0.001	1.250 (0.947–1.650)	0.113	0.566	0.145	0.592	0.836
ALP	1.034 (0.853–1.253)	0.731	0.812	0.732	1.223 (0.986–1.517)	0.066	0.566	0.069	0.043	0.373
GGT	1.113 (0.671–1.847)	0.675	0.812	0.725	1.244 (0.696–2.223)	0.456	0.919	0.497	<0.001	0.366
**B. Model 2**										
Fasting plasma glucose	1.109 (0.959–1.283)	0.160	0.283	0.129	0.967 (0.834–1.121)	0.651	0.992	0.525	<0.001	0.261
HbA1c	1.005 (0.870–1.161)	0.947	0.947	0.939	0.997 (0.860–1.155)	0.965	0.992	0.942	<0.001	0.375
LDL cholesterol	1.264 (1.048–1.523)	0.015	0.074	0.037	1.071 (0.877–1.308)	0.498	0.992	0.403	0.054	0.657
Triglycerides	1.332 (0.996–1.781)	0.053	0.134	0.103	1.002 (0.737–1.363)	0.987	0.992	0.989	0.130	0.519
AST	1.251 (1.009–1.550)	0.041	0.134	0.086	1.111 (0.889–1.390)	0.351	0.992	0.331	0.002	0.044
ALT	1.791 (1.368–2.344)	<0.001	<0.001	<0.001	1.243 (0.939–1.645)	0.127	0.635	0.161	0.580	0.830
ALP	1.013 (0.820–1.252)	0.900	0.947	0.908	1.235 (0.991–1.540)	0.060	0.601	0.063	0.022	0.109
GGT	1.167 (0.662–2.056)	0.589	0.842	0.664	1.220 (0.674–2.206)	0.507	0.992	0.539	<0.001	0.426

GMR, geometric mean ratio (MASLD/control or GA+AA/GG) from general linear models fitted to natural-log-transformed outcomes; Model 1 adjusted for age, sex, and genotype (MASLD comparison) or MASLD status (genotype comparison); Model 2 additionally for BMI. q, Benjamini–Hochberg false discovery rate-adjusted p value calculated across the 10 biochemical outcomes within each model and comparison; HDL cholesterol and total cholesterol were not log-transformed (their estimates in Tables 3 and 4 were used) but were retained in the correction family. p (HC3), p value based on heteroscedasticity-consistent (HC3) standard errors. SW, Shapiro–Wilk test of the log-scale residuals; BP, Breusch–Pagan test of the log-scale model.

Supplementary Table S2. Available and missing observations for the study variables, by group.

Variable	MASLD, available / missing	Control, available / missing	Overall available (% missing)
**Fasting glucose**	59 / 0	44 / 3	103 (2.8)
**HbA1c**	37 / 22	30 / 17	67 (36.8)
**HDL cholesterol**	53 / 6	42 / 5	95 (10.4)
**LDL cholesterol**	51 / 8	42 / 5	93 (12.3)
**Total cholesterol**	53 / 6	41 / 6	94 (11.3)
**Triglycerides**	52 / 7	42 / 5	94 (11.3)
**AST**	59 / 0	46 / 1	105 (0.9)
**ALT**	59 / 0	46 / 1	105 (0.9)
**ALP**	43 / 16	36 / 11	79 (25.5)
**GGT**	48 / 11	38 / 9	86 (18.9)
**Total bilirubin**	40 / 19	33 / 14	73 (31.1)
**Albumin**	46 / 13	37 / 10	83 (21.7)
**Ferritin**	43 / 16	36 / 11	79 (25.5)
**CRP**	32 / 27	36 / 11	68 (35.8)
**TyG index**	52 / 7	39 / 8	91 (14.2)
**Waist circumference**	59 / 0	46 / 1	105 (0.9)
**Hip circumference**	59 / 0	46 / 1	105 (0.9)
**Waist-to-hip ratio**	59 / 0	46 / 1	105 (0.9)
**Waist-to-height ratio**	59 / 0	46 / 1	105 (0.9)
**WWI**	59 / 0	46 / 1	105 (0.9)
**Fat-free mass**	59 / 0	46 / 1	105 (0.9)
**Body fat percentage**	59 / 0	46 / 1	105 (0.9)

Data were complete (n = 106; 59 MASLD, 47 controls) for age, body weight, height, BMI, 24-h energy intake, 24-h vitamin E intake, FFQ-derived vitamin E intake (mg) and adequacy (%), rs4988321 genotype (including the dominant-model recode), statin use, antidiabetic use, vitamin–mineral supplement use, and self-reported comorbidity. CRP, C-reactive protein; other abbreviations as in Tables 1–4.

Supplementary Table S3. Sex × MASLD interaction estimates for the anthropometric outcomes (exploratory analyses).

Outcome	n	Unadjusted interaction β (95% CI)	p	Age-adjusted interaction β (95% CI)	p
**BMI (kg/m²)**	106	1.90 (−1.99–5.78)	0.335	0.64 (−3.13–4.42)	0.735
**Waist circumference (cm)**	105	6.28 (−3.90–16.45)	0.224	2.32 (−7.14–11.77)	0.628
**Hip circumference (cm)**	105	1.43 (−6.12–8.98)	0.708	−0.23 (−7.73–7.27)	0.951
**Waist-to-hip ratio**	105	0.06 (0.00–0.11)	0.056	0.03 (−0.02–0.09)	0.222
**Waist-to-height ratio**	105	0.06 (0.00–0.13)	0.061	0.03 (−0.03–0.09)	0.281
**WWI**	105	0.88 (0.23–1.52)	0.008	0.52 (−0.01–1.05)	0.056
**Fat-free mass (kg)**	105	−2.57 (−7.60–2.46)	0.313	−1.86 (−6.94–3.23)	0.471

β, coefficient of the MASLD × sex interaction term (reference category: men); positive values indicate a larger MASLD–control difference in women. Mid-upper arm circumference was not available in the dataset.
